# Current and Future Approaches for Monitoring Responses to Anti-complement Therapeutics

**DOI:** 10.3389/fimmu.2019.02539

**Published:** 2019-11-08

**Authors:** Maedeh Mohebnasab, Oskar Eriksson, Barbro Persson, Kerstin Sandholm, Camilla Mohlin, Markus Huber-Lang, Brendan J. Keating, Kristina N. Ekdahl, Bo Nilsson

**Affiliations:** ^1^Division of Transplantation, Department of Surgery, University of Pennsylvania, Philadelphia, PA, United States; ^2^Rudbeck Laboratory C5:3, Department of Immunology, Genetics and Pathology, Uppsala University, Uppsala, Sweden; ^3^Centre of Biomaterials Chemistry, Linnaeus University, Kalmar, Sweden; ^4^Institute for Clinical and Experimental Trauma Immunology, University Hospital of Ulm, Ulm, Germany

**Keywords:** clinical trial, laboratory investigation, immunoassays, functional assays, CV%

## Abstract

Aberrations in complement system functions have been identified as either direct or indirect pathophysiological mechanisms in many diseases and pathological conditions, such as infections, autoimmune diseases, inflammation, malignancies, and allogeneic transplantation. Currently available techniques to study complement include quantification of (a) individual complement components, (b) complement activation products, and (c) molecular mechanisms/function. An emerging area of major interest in translational studies aims to study and monitor patients on complement regulatory drugs for efficacy as well as adverse events. This area is progressing rapidly with several anti-complement therapeutics under development, in clinical trials, or already in clinical use. In this review, we summarized the appropriate indications, techniques, and interpretations of basic complement analyses, exemplified by a number of clinical disorders.

## Introduction

### Physiological and Therapeutic Regulation of Complement Activation

The complement system consists of more than 50 soluble and membrane-bound proteins that function in both innate and adaptive immunity. Excessive complement activation contributes to the pathogenesis of many inflammatory diseases and numerous disease processes (summarized in Table 1). The underlying mechanisms include (1) increased and persistent activation, such as that resulting from the presence of soluble or solid-phase immune complexes as in systemic lupus erythematosus (SLE), myasthenia gravis, and related autoimmune disorders; (2) altered expression or function of various complement regulators as in paroxysmal nocturnal hemoglobinuria (PNH), atypical hemolytic uremic syndrome (aHUS), and C3 glomerulopathies (C3G); or (3) a combination of the two. Furthermore, complement activation is a part of reactions resulting from activation of all cascade systems of the blood, e.g., during ischemia reperfusion injury (IRI).

**Table 1:** Complement-related diseases and disease processes.

**Disease**	**Analyte**
Age-related macular degeneration	FH, FI, CD46 (MCP)
aHUS	FH, FI, CD46 (MCP)
Alzheimer's disease	C1q, C3, CR3
ANCA-associated vasculitis	C5a
Angioedema	C1-INH
C3 glomerulopathies	C3, FH, FHRs
Diabetic nephropathy	CD59
Encapsulated bacterial infection	C3
PNH	DAF, CD59
SLE	C1q, C1r, C4 or C2, FH, FCN3
Transplant	C3a, C5a, C5b-9, C4d

*MCP, membrane cofactor protein; FH, factor H; FI, factor I; aHUS, atypical hemolytic uremic syndrome; ANCA, anti-neutrophil cytoplasmic antibody; C1-INH, C1-inhibitor; FHRs, factor H-related proteins; PNH, paroxysmal nocturnal hemoglobinuria; DAF, decay accelerating factor; SLE, systemic lupus erythematosus; FCN3, ficolin 3*.

**Figure 1:**
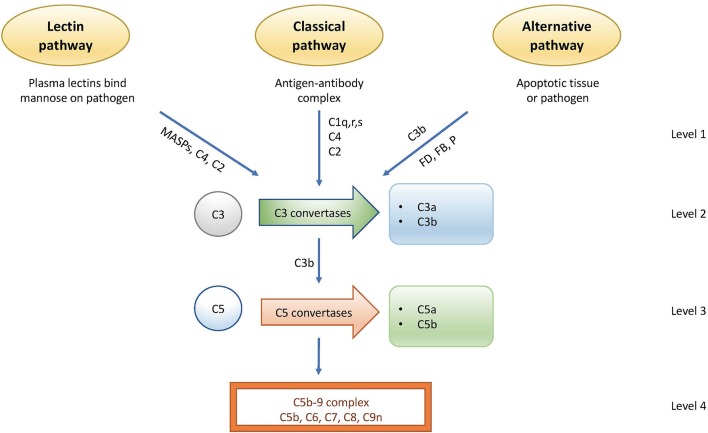
Activation and regulation of the complement system. Activation occurs via the lectin pathway (LP), the classical pathway (CP), and the alternative pathway (AP). Regulation occurs at distinct points. Level 1: inhibition of proteases generated by the LP and AP; level 2: control of the C3 convertases; level 3: control of the C5 convertases; level 4: control of the formation of the C5b-9 complex of the terminal pathway (TP).

The complement system is regulated at distinct levels as illustrated in Figure 1 and Table 2.

1. The first level of regulation occurs at the initiation level where the recognition molecules within two of the complement system's three activation pathways form complexes with proteases. In the classical pathway (CP), this complex is the C1 complex consisting of C1q, C1r_2_, and C1s_2_ molecules. In the lectin pathway (LP), the complex contains MASP-1 and/or MASP-2 and one of several recognition molecules, such as mannose binding lectin (MBL); Ficolin-1,−2,−3; or Collectin 10/11. All proteases in these pathways are regulated by C1 inhibitor (C1-INH), a serine protease inhibitor with broad specificity that also is active toward other cascade systems, such as the kinin/kallikrein system.
2. The second level involves the generation of C3 convertases that cleave C3 into C3a and C3b. The initiating complexes of the CP and the LP cleave C4 and C2 and generate the LP/CP convertase C4bC2a. In the alternative pathway (AP), a convertase is generated from C3, factor B, and factor D in conjunction with properdin, and in a self-perpetuating process, the AP convertase C3bBb is formed.
3. In the third level, the C5 convertases, which are derived from the C3 convertases, switch their specificity from C3 to C5, thereby cleaving C5 into C5a and C5b. The C5 convertases are regulated in the same way as the C3 convertases. C4b and C3b are regulated by the plasma protease factor I in three steps, mediated by one of several co-factors. The two first cleavages generate iC4b or iC3b, which lose their ability to generate either the C3 or the C5 convertases but which promote phagocytosis via interaction with complement receptors CR1 (CD35), CR3 (CD11b/CD18), CR4 (CD11c/CD18), and/or CRIg. The third factor I-mediated cleavage splits the molecule into the target-bound C4d and C3d,g (a ligand for CR2 or CD21) fragments and C4c/C3c, which is released from the activating surface.
  At this level, there are a number of regulators that protect autologous cell surfaces against complement attack. These include membrane-bound molecules, such as CR1, decay acceleration factor (DAF; CD55), and membrane cofactor protein (MCP; CD46), all of which disrupt the C3 convertases by different mechanisms (1). Additional regulators, including C4b-binding protein (C4BP, which regulates the CP/LP convertase) and factor H (the main regulator of the AP), are recruited from the plasma via glycosaminoglycans and/or deposited C3 fragments to the cell surface, thus providing further protection.
  The anaphylatoxins C4a, C3a, and C5a, which are generated by the cleavage mediated by C1s and the convertases, respectively, attract and activate mainly leukocytes via their receptors C3aR, C5aR1, and C5aR2. Recently, C4a was also shown to activate endothelial cells via the thrombin receptors PAR1 and PAR4 (2). Anaphylatoxins are regulated by carboxypeptidases (e.g., B and N) that desarginate the polypeptide in the C-terminus, leading to a significant, but not complete, loss of activity (3).
4. The final stage in the sequence is the formation of the C5b-9 complex (either the fluid-phase sC5b-9 or the membrane attack complex, MAC), which may insert into the cell membrane, thereby inducing cell lysis at high concentrations, or alternatively trigger inflammation and upregulation of tissue factor at sub-lytic concentrations (4). Terminal pathway (TP) regulators, such as cell-bound CD59 or vitronectin and clusterin in the fluid-phase regulate the formation and binding of C5b-9 to autologous cell surfaces.

**Table 2:** Regulatory targets of the complement system.

**Regulators**	**Function**
Carboxypeptidase-N	Removal of terminal arginine to degrade C3a and C5a
C1-INH	Inhibits C1r, C1s, MASPs
C4BP	Accelerates decay of LP/CP convertases Cofactor for FI
CD46 or MCP	Cofactor for FI
CD55 or DAF	Accelerates decay of convertases
CD59 or Protectin	Binds to C8 and C9, prevents assembly of terminal complement complex
FH	Recognizes self surfaces, accelerates convertase decay, cofactor for factor
FHL-1	Accelerates convertase decay, cofactor for factor I
MAP-1	Binds to MBL/ficolins, inhibits C4 deposition
Type 1 complement receptor (CD35/CR1)	Dissociation of C3 convertase subunits, cofactor for factor I-mediated cleavage of C3b and C4b

*C1-INH, C1 inhibitor; MASPs, mannan-binding lectin serine proteases; C4BP, C4 binding protein; FI, factor I; MCP, membrane cofactor protein; DAF, decay accelerating factor; FH, factor H; FHL-1, factor H like protein 1; MAP, MBL/ficolin associated protein; MBL, mannose-binding lectin*.

### Mechanisms of Therapeutic Regulation

Substances intended for therapeutic regulation of complement in human disease have a number of different mechanisms of action (5). Antibodies against functional sites in the molecule are commonly used and best exemplified by eculizumab and ravulizumab, which are antibodies that prevent the cleavage of C5 by the C5 convertases. Alternatively, small molecules, aptamers (6), or peptides are used, which can either block the active site of serine proteases or prevent the interaction of proteins in the complement cascade. Examples in this class of complement inhibitors include factor D inhibitors and peptides of the compstatin family that prevent the substrate C3 to be cleaved by particularly the AP C3 convertase, as well as the CP C3 convertase. A final group and probably the largest one consist of recombinant proteins in either full-length or truncated forms of physiological regulators of complement. The first example is soluble complement receptor (sCR1 [CD35]; TP10), which is a receptor and regulator of the convertases that acts as a cofactor for factor I, or by increasing the decay of the convertases. CR1 belongs to a large family of complement regulators, which consists of varying numbers of homologous domains, so-called short consensus repeats (SCR). CR2 (CD21), MCP (CD46), DAF (CD55), factor H, and C4BP are found in this family. Many of those, in full-length or in truncated recombinant forms, have been engineered to regulate complement activation for therapeutic purposes. Serpins, such as C1-INH, are another type of regulators with broader specificity and have been employed as therapeutics. Purified or recombinant C1-INH is one of two complement-targeting drugs (together with anti-C5; eculizumab and ravulizumab) that have been approved for clinical use.

Antisense strategies to silence the gene expression of a drug target are a more recent development in drug discovery, where targeting at the DNA or RNA level may be a way to overcome high target concentrations. This strategy has also been applied to the complement field. Alnylam Pharmaceuticals has developed a C5-directed RNAi therapeutic that is liver-targeted through GalNAc conjugation and silences intrahepatic expression of C5 (5, 7). Hence, this strategy leads to a systemic reduction in C5 levels and terminal pathway activity, and is currently being trialed as a therapy for PNH and aHUS. Likewise, the company Ionis Pharmaceuticals has developed a systemically administered factor B targeting antisense oligonucleotide that has also entered clinical trials with the aim of reducing AP activity in AMD and IgA nephropathy (5, 8). While some antisense therapies are directed systemically, this strategy also offers an opportunity to target complement factors in a specific tissue or at the site of disease through delivery systems, such as antibodies.

## Analytical Methods

Robust and accurate measurement of complement proteins and activation products is required, in order to monitor patients treated with drugs that result in detectable changes in the complement status, such as treatment with compstatin variants or anti-C5. Standard complement evaluation includes three main categories: (1) complement function, (2) quantification of individual complement protein, and (3) quantification of activation products (9).

### Pre-analytical Handling and Methodological Considerations

**Figure 2:**
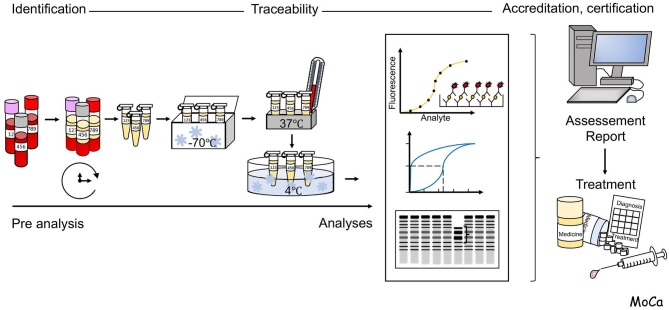
Schematic overview of the flow of events in the process for analysis of clinical patient samples. Reliable analysis of samples requires the control of pre-analytical handling of and choice and validation of appropriate analytical techniques, Detailed information is given in the section “Pre-analytical handling and methodological considerations”.

A schematic overview of the process for analysis of clinical patient samples is illustrated in Figure 2.

A major issue to take into consideration for laboratory analysis of complement activation is the choice of biological sample and, if applicable, the use of anticoagulants. EDTA-plasma is suitable for quantitation of individual complement factors and for assessment of activation products, while serum is suitable for analysis of complement function. Serum can be substituted by plasma that is anticoagulated with FXa or thrombin inhibitors (e.g., Dabigatran, or lepirudin, i.e., recombinant hirudin, respectively), which do not disturb complement function. As a general recommendation, the samples should be frozen at −80°C within 120–240 min. It is important not to freeze the specimen at −20°C, which creates a slow freezing rate and further activation/inactivation of individual components. The optimal transportation method is using dry ice containers. In addition, measurement of split products with enzyme-linked immunosorbent assay (ELISA) requires cautious handling, such as collecting the blood in EDTA-containing tubes, immediately placing samples on ice, and storage at −80°C after 30 min of centrifugation. The issue of sample handling in relation to generation of complement activation *in vitro* has been discussed extensively elsewhere [e.g., (9–11)].

### Functional Assays

The proper functioning of the individual complement activation pathways depends on the integrity of each of its participating component(s), and therefore a functional test that monitors a whole activation pathway from initiation to the effector phase (formation of the C5b-9 complex, i.e., lysis) can detect both therapeutic-induced deficiencies in complement components and consumption-related decreases in complement activity, thus combining the information obtained from the various types of assays described above.

Traditionally, complement function by the CP is assessed by hemolytic assays that use sheep erythrocytes coated with rabbit antibodies (preferably IgM but sometimes combined with IgG). When serum (or lepirudin-anticoagulated plasma) is added, C1q binds to the immunoglobulins, leading to the assembly of the C5b-9 complex of the terminal pathway, thereby lysing the sheep erythrocytes (12, 13). Complement activation by the AP is monitored by the same assay principle with the exception that rabbit or guinea pig erythrocytes are used instead, as these are spontaneous activators of the human AP.

Hemolytic assays can be performed in various ways; the original assays, the so-called CH50 and AH50 assays, are based on titration of the amount of serum needed to lyse 50% of a fixed limited amount of cells during a certain time interval (12, 14). The considerably less laborious, and more rapid, one-tube assays give similar results and is based on the fact that the “dose” of complement is proportional to the number of cells lysed and the assay is therefore performed in an excess of erythrocytes (12).

Hemolytic assays are quite sensitive to the specific individual-derived erythrocytes that are used in the assays. Probing the erythrocytes before use is necessary in order to choose the right preparation. Most functional assays are linear in their dose-response except for the functional ELISAs, since there is no standard curve applied in these assays.

As an alternative to erythrocytes, liposomes coated with an activator are used in some tests and the assays are otherwise performed in a similar manner to CH50 assays. An important advantage with using artificial liposomes as activators is that results are no longer dependent on the source of animal of the RBCs used, which should improve reproducibility over time.

More recently, a method was introduced that made use of three separate ELISAs, for the first time enabling the simultaneous determination of all three activation pathways (including the LP). The assay can best be described as a “solid-phase functional test,” since it incorporates recognition structures specific for each pathway (IgM for the CP, mannan or acetylated bovine serum albumin [BSA] for the LP, and LPS for the AP). These molecules are coated onto microtiter plate wells, and then serum is added and incubated under conditions in which only one pathway is operative at a given time, and the other two pathways are blocked. For each ELISA, the final step is the detection of the resulting C5b-9 complex by monoclonal antibodies (mAbs) against a neo-epitope in complex-bound C9 (15). One can here expect that the assay for AP activation will differ from the hemolytic assays in that the ELISA depends on LPS activation and properdin while the hemolytic assay lyse the cells because of an insufficient regulation of the AP on the cell surface.

### Individual Components

The concentration of individual complement proteins is determined by various quantitative immunoassays. The most common employed methods are immunoprecipitation techniques, today mainly nephelometry and turbidimetry, where polyclonal antibodies against the protein of choice, e.g., C1-INH, C4, C3, or factor B, are added to the sample to form immune complexes that will distort the detection of light beams passed through the sample. Turbidimetry measures antigens based on changes in the light transmission. These techniques are accurate and fast, and have a large capacity and low variance. Also, C1q is commonly analyzed by nephelometry but is an inappropriate analyte for this technique due to its antibody binding properties (16). However, one of the main challenges for these methods is antibody reactivity with breakdown components or parent proteins particularly in C3, factor B, and C4 assays. For components with a low plasma concentration, ELISAs are more appropriate (17). This technique is also applied in measurement of activation products and autoantibodies against complement factors.

Recently, multiplex assays for complement components have been introduced and are now commercially available. The advantage of such assays is the simultaneous determination of several components, thereby saving both time and sample volume. To date, the analytes in the available kits have been restricted to components with high plasma concentrations, and no LP-specific panels are available yet on the market. There is no standardized regulatory guideline for validation of these tests. On the other hand, cross reactivity of reagents and inter- or intra- CV% are challenging aspects of multiplex immunoassays (18).

### Activation Products

Complement activation generates activation fragments and protein–protein complexes, which can be quantified to assess the magnitude of complement activation. Two principles are used in assays for activation products: (1) mAbs specific for epitopes that are hidden in the native protein but exposed upon activation (so-called neo-epitopes). Most available assays for C3a, C3b/iC3b/C3s, C4a, C4b C4d, Ba, Bb, and aC5b-9 are based on mAbs to neo-epitopes (15, 19). A potential issue is that supposedly neoepitope–specific antibodies are polyreactive and also recognize the native protein. Even a low level of cross-reactivity can disturb the assay as the native protein typically is present in a much higher concentration than its activation product. This is the case using mAbs against C4d for monitoring CP activation in the fluid phase or in biopsies that also detect C4b, iC4b, as well as intact C4 (20). (2) The other option is to use polyclonal antibodies that often require fractionation of the native protein from its activation fragment or product. This principle is used for quantification of C3d,g by nephelometry. Polyclonal (and monoclonal) antibodies can also be used in sandwich ELISAs for quantitation of protein complexes, such as C1s-C1-INH, C3bBbP, sC5b-9 complexes, etc. The aforementioned assays are based on immune reactivity and antibody specificity.

### Complement Analysis in Tissues or Body Fluids Other Than Serum/Plasma

In addition to analyses of plasma/serum, which measures systemic complement activation, it may, in many cases, be more informative or even required to determine the local activation state in a particular compartment or tissue. Increased levels of complement activation products during inflammatory processes can be found, e.g., in body fluids, such as cerebrosinal fluid (CSF) (21) or synovial fluid. Here, the assays outlined above can generally be used, whereas the rate-limiting factor may be the availability of material as sampling is more complicated compared to a peripheral venipuncture.

Lastly, staining of tissue sections or biopsy material for deposition of complement activation products will give information of the degree of complement activation at the local site of the organ. This is performed in clinical routine for, e.g., complement-mediated glomerulopathies.

A potential future methodology not yet in clinical practice would be to combine non-invasive imaging methods with a complement specific probe, e.g., a neo-epitope specific antibody coupled to a tracer. This method has been applied to antibodies specific for the C3 activation products C3d,g, with a recent example of an animal model of tuberculosis infection (22).

### Analysis of Complement-Induced Receptor Signaling

The anaphylatoxins C3a and C5a, formed as a result of an activating cleavage of C3 and C5, respectively, are potent biological molecules that exert diverse biological effects on cells and tissues via their cognate receptors. The activating C5a receptor C5aR1 (CD88) is the target of ongoing drug development programs, where ChemoCentryx has a small-molecule inhibitor (Avacopan/CCX168) in trials for aHUS, ANCA-associated vasculitis, and hidroadenitis suppurativa (23). Using an alternative strategy, Innate Pharma has developed a C5aR1 blocking antibody (IPH5401) currently in a phase I study as an adjuvant to cancer immunotherapy, testifying to the diverse biological effects of C5a signaling (24). To directly assess C5aR1 receptor blockade by CCX168, Bekker et al. performed an *ex vivo* assay after drug administration to healthy volunteers, with inhibition of CD11b upregulation on circulating neutrophils by exogenous C5a as endpoint (25). Such an *ex vivo* assay design will likely also be of value when designing assays to evaluate blockade of the receptor for C3a, which currently are in preclinical development (26) and may hold promise as a drug to enhance the efficacy of cancer immunotherapy (27).

### Performance of Various Techniques

Most assays have either high specificity and low sensitivity or low specificity and high sensitivity. The conclusion is that one often needs more than one assay for diagnosis of a disease, i.e., determination of the degree of activation and which pathway that is activated.

Of the techniques above, most are easy to perform and can be used on many samples. One exception is the CH50 and AP50, which require serial dilutions of the samples, and if low levels are obtained, there may be a requirement that the samples must be reanalyzed (13, 14). Other assays are therefore warranted.

The coefficient of variation (CV%; standard deviation/mean value) is an extremely important property of an assay, which affects the power of the trial, i.e., the probability to reject a false null hypothesis. High CV% is associated with poor discrimination between various populations, while a low CV% will allow the opposite and is associated with a successful trial. The CV can be calculated either as an intra-assay (within one run) or inter-assay (between several runs) CV. The intra-assay CV is relevant in small trials when all samples can be run at the same time in one batch. If the samples are analyzed consecutively over time, the inter-assay CV is of great importance, since it will reflect differences between different runs performed on different days. For instance, one study demonstrated an intra-assay CV% for alternative pathway components ranging from 3% (factor D) to 8% (factor B) and inter-assay CV% ranging from 5% (factor D) to 15% (factor B) (28).

The CV% depends on the pre-analytical handling, the type of assay that is used and the performance of the laboratory. When considering the CV%, it can be as low as 5% or less when nephelometric or turbidimetric assays are applied. For other immunochemical assays including ELISAs, 10% and more are common. The CH50 and AP50 are poised to have high CV% if they are not run in the same batch of tests, due to the inborn variation of the assay and the different erythrocyte preparations. The one-tube hemolytic assay is much better in this respect and has a CV of <10% in the normal range (12) (Nilsson B, unpublished data). The intra-assay CV% for alternative and classic pathway function were also determined by more advanced ELISA techniques as 3.2 and 5.7%, respectively (29).

A laboratory may also have more than one device to analyze the samples and here it is important to make sure that these devices have the same precision and variation. Also, it is not recommended to use more than one laboratory for sample analysis to limit additional confounding from site to site. There is only one international standard (the IFCC international reference preparation [CRM470]) that is available for C4 and C3. Despite this, there is a huge difference in the precision between different turbidimeters and nephelometers (30, 31). Despite accurate measurement methods, it is usually difficult to distinguish between the exact activation mechanism by only measuring activation products, since more than one pathway is often involved in the activation in many conditions (32).

To illustrate this, a group of researchers compared three different assays to measure immune complexes by ELISA, including C1q binding assay, deposition in solid phase C3 binding glycoprotein (CIF) and anti-C3 antibodies in sera from patients with SLE, rheumatoid arthritis, and systemic sclerosing. All three tests showed specificity over 95%, but various sensitivity (C1q-ELISA−28.97%, CIF-ELISA−19.63%, anti-C3-ELISA−17.29%), which indirectly affects the correlation coefficient for each disease category (33).

### Emerging Techniques for Measuring Complement Function, Components, and Fragments

The advent of the Human Genome Project greatly accelerated subsequent developments in transcriptomics, proteomics, metabolomics, and other “omic” technologies (34). Platforms to study genome-wide DNA variants have advanced from genome-wide genotyping arrays targeting hundreds of thousands to millions of single-nucleotide polymorphisms (SNPs) to whole exome sequencing (WES) and whole genome sequencing (WGS) with wet-lab and analyses pipelines as well as pricing continuing to improve. Transcriptomics has advanced from array-based RNA/cDNA nucleic acid probes for genome-wide RNA expression studies, to “whole-transcriptome” sequencing of RNA molecules (often termed bulk “RNAseq”) to single-cell RNAseq where RNAs can now be interrogated at single-cell resolution [reviewed in (35)].

Proteomic and metabolomic approaches often overlap in sample preparation techniques and separation of molecules by mass/size, charge, and hydrophobicity. The analytic processes and platforms have also advanced significantly on a number of fronts including sample preparation processes, chemistry, and analytical instruments (36–38). One of the most commonly used analysis pipelines uses liquid chromatography and mass spectrometry (LC-MS) of peripheral blood leukocytes, blood/plasma/serum, or CSF preparations in which the proteins have been digested with trypsin into peptides. Peptides from multiple time points are typically tagged using isobaric mass tags (TMT labeling) in order to perform relative quantification (36, 38, 39).

Tagged peptides from each time point are often separated using a reverse-phase HPLC gradient directly coupled to a standard mass spectrometer instrument. Peptide sequences can be determined by tandem mass spectrometry (MS/MS) coupled to database searching, such as the Sequest algorithms and Proteome Discoverer software. The peak intensity for each identified peptide across all experimental/clinical time points can then be calculated from the LC-MS data. Integrating genome-wide genotyping or sequencing with transcriptomics, as well as proteomic, metabolomic, and other omic datasets from blood and other tissues from samples of individuals selected for phenotypes of interest and appropriate controls over extended time periods can be performed using approaches such as integrative Personal Omics Profiling (iPOP). Such longitudinal multi-omic iPOP studies over prospective health and disease time points have revealed major insights into dynamic biological processes including multiple significant infections, and the development of a range of diseases (36, 38, 39).

A number of specific considerations should be made when analyzing such studies in the cascade characteristics of the complement system. The peripheral, dynamic, and temporal nature of the complement cascade means that in order to maximize insight, samples have to be taken at the most appropriate time points—and special consideration needs to be paid to sample storage and preservation as these platforms are particularly sensitive to oxidation—so samples need to be stored rapidly in liquid nitrogen or at −80°C with care to displace oxygen for long-term storage using liquid nitrogen or argon. Multiomic analysis can be performed to discover “omics” signatures related to complement related primary or secondary outcomes, using approaches such as ANOVA-based differential methods (40) or more complex machine learning methods. Sets of genes, proteins, metabolites, and other omic datasets can be tested for enrichment in *a priori* defined molecular pathways using standard bioinformatics tools (41).

Proteomic-based approaches have been used to investigate the potential involvement of complement in numerous human diseases and pathological conditions, both systemic and involving different specific organs. Here, we present a few examples from a rapidly growing body of analytical data.

The complement system plays an important role in the protection of the eye and complement components and regulators have been identified in most parts of the eye, using immunohistochemical, mRNA-based, or, more recently, proteomic approaches. However, dysregulated complement activation is implicated as a driving force in a number of ocular diseases, mostly studied in age-related macular degeneration (AMD), but also in glaucoma, uveitis, and neuromyelitis optica as reviewed in (42).

Formation of extracellular retinal deposits called drusen along Bruch's membrane in the submacular space is a risk factor for developing AMD, but drusen is also found in non-AMD individuals. Unbiased proteome analysis by LC-MS iTRAQ (isobaric tags for absolute quantification) technology of human drusen has identified 129 proteins, out of which one third were found only in AMD. Complement components that were elevated in AMD included C3, C5, C6, C7, C8γ, C9, and the regulators vitronectin and clusterin (43, 44).

Immune defense proteins, including complement proteins, were also quantitated in the macular Bruch membrane/Choroid complex in a study of human post-mortem eyes comprising 24 AMD eyes (10 early/mid stage, six advanced dry AMD, and eight wet AMD) and 25 normal control eyes. A total number of 901 proteins were identified, most of which did not differ in concentration between AMD and controls and were therefore concluded to reflect the proteome of normal macular tissue at the age of 81 years (the average age of the eye donors included in the study). Fifty-six proteins were increased and 43 decreased in AMD compared to controls. Approximately 60% of the elevated proteins were related to immune response and/or host defense, including C3, C4, C5, C6, C7, C8a, C9, factor B, factor D, and the regulators factor H and clusterin (45). The elevated protein constituent has proven to be variable in different stages of AMD, which is indicative of various mechanisms of disease progression, suggesting that a tailor-made complement-modulating treatment is needed (45).

The complement system also has a known role in diabetes and related complications. A recent iPOP study assessed the early biological processes impacting the transition to clinical type 2 diabetes (T2D). Multiomic profiling from healthy and prediabetes individuals (*n* = 106 total) took place over 4 years. Extensive host and microbial changes were observed to occur during respiratory viral infections, and insulin-resistant participants responded differently from insulin-sensitive participants. Furthermore, specific host–microbe interactions were observed to differ between insulin-resistant and insulin-sensitive individuals. Interestingly, these xenobiotics were also tightly associated with expression of host factors involved in the complement system (C4B, C4BPB, and C4BPA), which is associated with the development of diabetes (38).

The Integrative Human Microbiome Project (iHMP) followed 100 adult pre-diabetic participants for several years (46) and subjected a subset of 23 individuals to weight perturbation, where they consumed an additional 1,000 kcal per day for 30 days. All individuals were subjected to WES at baseline and multi-omic profiles were generated at all time points comprising RNAseq, proteome LC-MS of PBMCs, Proseek multiplex analytes from plasma, metabolomics (LC-MS), circulating cytokines (Luminex), and 16S rRNA sequencing and whole metagenome shotgun sequencing of microbiota. These data represent the largest integrative profiling project ever conducted on a cohort of humans. Despite the modest weight gain induced in this perturbation study, a wealth of biomolecular changes was evident across omic data types. Integrating proteomic and transcriptomic information revealed significant differences between pre-diabetics and healthy controls even at baseline, with many of these indicative of autoimmune responses (37). After weight gain, participants showed significant changes in pathways related to inflammation including complement pathways.

Additional studies looking at more limited combinations of omics have been attempted in complement-related diseases. These included combining transcriptomics and genotyping datasets in abdominal aortic aneurysms (AAAs) (47). Additional integration of expression data from bladder, breast, colon, lung, and lymphoma cancers with genomic datasets from the same individuals have also been investigated and have revealed that Complement C1q -Binding Protein (C1QBP) showed association with patient survival, and identified C1QBP-involved pathways as potential targets for therapeutic intervention (48).

Defining the characteristics of the complement system in an individual can also help tailor individualized treatment with complement-targeted therapeutics. A well-known example that illustrates this concept well is the case of eculizumab resistance, where the genetic basis underlying a poor response to the drug was originally described in Japanese PNH patients (49). It was shown that poor responders harbored a C5 coding polymorphism that abrogated eculizumab binding while C5 still retained hemolytic activity. The mutation was subsequently confirmed to coincide with the eculizumab binding epitope in C5 (50). Akari therapeutics has launched clinical trials with the tick-derived peptide Coversin that blocks C5 cleavage via a binding site that is non-overlapping with eculizumab. Notably, Coversin is explored as a treatment option in patients with proven eculizumab resistance (51). This being one particularly illustrative example, next-generation sequencing methods or other omics techniques will undoubtedly be valuable in identifying other variants in an individual's complotype that limit or influence the response to a complement-targeting drug.

## Therapeutic Regulation of Complement in Human Disease

### Examples of Therapeutic Complement Regulators

Currently, there are only two types of complement inhibitors available in the clinic: C1-INH preparations and C5-targeting antibodies. C1-INH, either purified from plasma or produced in recombinant form, inhibits proteases generated by the CP and LP (C1r, C1s, MASP-1, and MASP-2) in addition to FXIIa, FXIa, and KK, which are activated by the contact system. The clinical use of C1-INH preparations is as substitution therapy in hereditary angioedema, and not as a complement inhibitor *per se*, although this possibility is explored in extension trials with, e.g., transplantation as indication. Eculizumab is a humanized anti-C5 mAb that prevents activation of C5 and thereby both the generation of C5a and the initiation of C5b-9 formation. Indications for eculizumab are aHUS, PNH, and refractory myasthenia gravis, and it is currently in clinical trial for, e.g., ABO-incompatible kidney transplantation. Ravulizumab is a further development of this antibody that has a more prolonged half-life (52).

In addition to these two inhibitors, a large number of compounds that act at different control points within the complement cascade are under development for various indications. Some compounds that are currently in clinical trials are anti C1s mAbs that inhibit CP activation, peptides within the compstatin family that all bind to C3, thereby making it resistant against activation by both C3-convertases, and APT070, which blocks downstream complement activation by inhibiting the C3-convertases. An intriguing example is Omero's inhibitory antibody to the LP protease MASP2, currently in trials as a treatment for aHUS, which has been considered a prototype of an AP-driven disease (Table 3).

**Table 3:** Monitoring of complement activity in clinical trials of complement therapeutics.

**Target**	**Drug candidate**	**Company**	**Entity**	**Indication**	**Status**	**Assay for monitoring complement activation**	**References**
C1r, C1s, and MASPs	Cinryze	Shire	Protein	Transplantation	Phase I	Classical pathway and MBL pathway activity	(53)
	Cetor	Sanquin	Protein	Trauma or sepsis	Phase III	C1 inhibitor concentration	(54)
	Ruconest (conestat alfa)	Pharming	Protein	Contrast-induced nephropathy	Phase II	C1 inhibitor serum levels	(55)
C1s	BIVV009	Bioverativ	Antibody	Cold agglutinin disease	Phase 1	Classical pathway Wieslab® assay CH50	(56)
MASP2	OMS721	Omeros	Antibody	Thrombotic microangiopathies	Phase II	Lectin pathway activation	(57)
				aHUS	Phase III	C3 activity C4 activity	(58)
C3	AMY-101	Amyndas	Peptide	C3G	Phase 1	CH50 AH50 C3 plasma levels C4 plasma levels	(59)
	APL-9	Apellis	Peptide	PNH	Phase 1	CH50 AH50 C3 serum levels C3a serum levels	(60)
FD	Lampalizumab	Genentech	Antibody	AMD and/or GA	Phase III	Complement factor I profile biomarker (genotype)	(61)
	ACH-4471	Achillion	Small molecule	PNH	Phase II	Alternative pathway Wieslab® assay Factor D Factor Bb	(62)
FB	LNP023	Novartis	Small molecule	C3G	Phase II	Circulating C3 levels Circulating Bb levels Circulating sC5b9 levels	(63)
				PNH	Phase II	C3 fragment deposition on RBCs	(64)
				PNH	Phase II	C3 deposition on RBCs	(65)
				C3G	Phase II	C3 deposit score in kidney biopsies C3 levels Bb levels	(66)
				IgA nephropathy	Phase II	Bb levels sC5b-9 levels	(67)
Convertases	Mirococept	MRC	Protein	Transplantation	Phase III	Complement activity in serum C3a levels in urine	(68)
C5	Soliris (Eculizumab)	Alexion	Antibody	Membrane proliferative glomerulonephritis	Phase II	sC5b-9 levels	(69)
				Guillain-Barré syndrome	Phase II	Hemolytic complement activity in serum	(70)
				STEC-HUS	Phase III	CH50	(71)
	Tesidolumab (LFG316)	Novartis and MorphoSys	Antibody	AMD and/or GA	Phase II	C5 concentration in blood	(72)
				Uveitis and/or panuveitis	Phase II	C5 serum levels	(73)
	SKY59 (RG6107, RO7112689)	Chugai and Roche	Antibody	PNH	Phase I/II	*Ex vivo* liposome lysis in serum C5 serum levels	(74)
	REGN3918	Regeneron	Antibody	PNH	Phase I	CH50	(75)
	ABP959	Amgen	Antibody	PNH, aHUS	Phase I	CH50	(76)
	Coversin	Akari	Protein	PNH	Phase II	CH50 ELISA	(77)
	Cemdisiran	Alnylam	Oligonucleotide	PNH	Phase I/II	Complement activity in serum C5 serum levels	(78)
C5a	IFX-1	InflaRx	Antibody	Sepsis	Phase II	C5a plasma levels	(79)
				SIRS, complex cardiac surgery	Phase II	C5a plasma levels CH50	(80)
				Hidradenitis suppurativa	Phase II	C5a plasma levels	(81)
C5aR1	Avacopan (CCX168)	ChemoCentryx	Small molecule	aHUS	Phase II	C3 serum levels C4 serum levels C5 serum levels Factor H C5a *Ex vivo* C5b-9 deposition on microvascular endothelial cells	(82)

*Adapted from Ricklin et al. (83). Only trials with specified methodology for complement monitoring were included*.

*AAV, anti-neutrophil cytoplasmic-antibody-associated vasculitis; aHUS, atypical hemolytic uremic syndrome; ABOi, ABO incompatible; AMD, age-related macular degeneration; APS, antiphospholipid syndrome; CNV, choroidal neovascularization; COPD, chronic obstructive pulmonary disease; GA, geographic atrophy; GVHD, graft vs. host disease; IPCV, idiopathic polypoidal choroidal vasculopathy; LN, lupus nephritis; MN, membranous nephropathy; PNH, paroxysmal nocturnal hemoglobinuria; SIRS, systemic inflammatory response syndrome; wAIHA, warm autoimmune hemolytic anemia; CH50, classical pathway hemolytic assay; AH50, alternative pathway hemolytic assay; sC5b9, soluble C5b9 complex*.

Drug candidates in preclinical development are also expanding the potential use of these drugs beyond “classical” complement-driven diseases to neurological disorders like multiple sclerosis and Alzheimer's disease. There are a number of ongoing Phase I, II, and III clinical trials across a variety of disease spectra reviewed in detail [e.g., (83, 84)].

### Principles for Monitoring the Effect of a Therapeutic Drug *in vivo*

Monitoring of the effects of complement therapeutics can be achieved using assays described above, measuring either the functional capacity of a certain pathway or the circulating levels of a specific component or activation product. When a complement component is activated *in vivo* either by proteolytic cleavage and/or other types of conformational changes triggered by, e.g., protein–protein interactions, the individual component is taken up by cells, e.g., leukocytes and Kupffer cells, leading to decreased levels of the component. This results in decreased levels (consumption) of complement components. If a whole pathway (CP + TP or AP + TP) is activated, all components are consumed along the activation sequence, and the function is reduced in the affected pathway while activation products derived from the individual affected components will be increased. If a therapeutic drug affects the complement activation at the level of an individual component, then the specific component and the downstream complement factors will be affected.

We conducted a systematic survey of registered clinical trials of complement-targeting drugs (83) to illustrate how complement activation is monitored during the clinical phase of drug development. Thirty trials were found where a biochemical measure of complement activation was among the endpoints (Table 3). Generally, in trials where information on complement monitoring was available, patient samples (typically serum) were subjected to well-established assays, such as CH50/AH50 or functional ELISA. When combined with measurements of activation products and individual complement components, these assays provide a fair assessment of the complement inhibitory activity of a drug *in vivo*. However, as outlined above, drawbacks in current methods for complement testing include limited sensitivity and susceptibility to errors in sample handling. We argue that novel methods could be of value, in order to precisely probe the efficacy and specificity of a drug and understand the physiological consequences of complement inhibition. In addition, determining a patient's complement-related genotype prior to starting a new drug may help to individualize treatment and select the complement-targeting drug that is most likely to be efficient in a given individual. Such improvements will in turn aid investigators in the development of the coming generations of complement therapeutics. Likewise, improved methods are needed to achieve a deeper physiological understanding of the consequences of complement inhibition in patients.

In this context, it is also important to note that drug levels and by-products should be quantified for pharmacological and toxicological purposes. Each drug requires a specific assay that can be based on various techniques, e.g., immunoassays (proteins, antibodies), HPLC, mass spectrometry, etc. In the case of eculizumab, quantification of the specific antibody is possible by ELISA (29). However, two populations of antibodies are circulating, one in complex with C5 and one that is not bound to the antigen (due to the long half-life). Two assays are necessary if one wants to keep track on both populations. The antibodies not bound to the antigen can be detected using a direct-binding assay with solid-phase bound C5 detecting bound IgG, while the other assay needs to pull down C5–IgG complexes using anti-C5 (other epitopes than eculizumab) and detecting IgG. Using these assays, it is possible to follow the pharmacokinetics of the drug.

## Discussion

A large number of clinical trials evaluating complement regulatory drugs have been completed or are currently ongoing. It has become evident that there is a pressing need to improve monitoring of the complement status in patients receiving treatment with these drugs; a need that is only expected to increase in the future given the extensive list of complement-targeting drugs that are in clinical trials. Poor laboratory assessments can obviously lead to inconclusive results, and the limitations of the currently available complement assessments are the CV of the assays and the selection of the complement specific tests. The CV results from not only the specific test that is used but also the pre-analytic handling of the samples before analysis. All these parameters are under the influence of the laboratory that performs the analyses.

As indicated above, the samples need to be processed as soon as possible (preferably in 4 h or less) including centrifugation at +4°C and storage at −80°C to avoid damage to the sample, particularly if the analyte is an activation product. Transportation should be on dry ice after freezing at −80°C. For instance, some of the split products have very short half-lives, making robust reproducible measurements very difficult. Poor handling will inevitably lead to variations. Also, the assay selection is of great importance since the difference in CV can be as much as 5–10 CV% between different assays. For instance, the difference between CH50 and a functional ELISA of the CP or the one-tube assay can be substantial, with CV% up to 20% often observed, particularly if inter-assay coefficient variations are being considered. Similar differences in CV% can occur if the protein concentrations are assessed by either radial immunodiffusion or nephelometry. The variation will lead to poor discrimination between individual comparisons as well as wide reference intervals and therefore blunt discrimination between normal and pathological values. So which level of CV% is acceptable? This is totally dependent on the parameter that is supposed to be evaluated. The lowest possible level is recommended since high CV% will lead to less power in the trial and the need of additional test individuals.

What is the desired or appropriate level of complement inhibition to seek with therapeutics in order to achieve a clinical response? Targets within the complement system are often plasma proteins with high circulating concentrations, which in turn necessitate high drug concentrations. A well-described phenomenon in PNH patients during anti-C5 therapy with eculizumab is breakthrough hemolysis. Defined as either pharmacokinetic or pharmacodynamic breakthrough, it results from insufficient dosing or massive complement activation exceeding the inhibitory capacity of eculizumab. To overcome this problem, there are strategies to increase the half-life of C5 antibody preparations, e.g., to promote its recycling from endosomes as in the case of Alexion's ALXN1210 (Ravulizumab). Several trials also evaluate additional C5 targeting drugs as add-on therapy to eculizumab. Hence, a very high degree of inhibition appears to be necessary to completely block complement-mediated hemolysis in this condition; on the other hand, the clinical benefit of complete complement inhibition remains controversial. For example, a low degree of residual intravascular hemolysis may not be considered be a clinically relevant issue (85).

The term “complotype” has been coined to describe an individual's genetic setup of common complement polymorphisms that determine complement activity on the genetic level (86). Assessing the genetic basis for complement activity in an individual, e.g., with targeted genotyping or next-generation sequencing, will undoubtedly facilitate individual dosing of complement-targeting regimens. Another potential implication is that the minimal required degree of inhibition may vary between patients depending on the genetic setup of their complotype.

In order to get a full view of the effect of an anti-complement drug, many times not only one parameter is sufficient. Analysis of all three complement categories (function, single component, and activation product) is necessary in order to get the full picture. With the advent of novel omics analyses and multiplex assays, detailed analyses of individual complement components and activation products are possible. This will help to follow pharmacokinetic events and possible side effects. It will also allow detailed analyses of other types of side effects, such as metabolic changes by metabolomic analyses.

## Author Contributions

MM, OE, BK, KE, and BN wrote the article. MM and CM prepared the figures. BP, KS, and MH-L edited the manuscript. All authors approved the final manuscript.

### Conflict of Interest

The authors declare that the research was conducted in the absence of any commercial or financial relationships that could be construed as a potential conflict of interest.
